# Modified lateral pressure formula of shallow and circular silo considering the elasticities of silo wall and storage materials

**DOI:** 10.1038/s41598-022-11305-6

**Published:** 2022-04-29

**Authors:** Zhijun Xu, Pengfei Liang

**Affiliations:** Henan Key Laboratory of Grain and Oil Storage Facility & Safety, Henan University of Technology, Zhengzhou, 450001 China

**Keywords:** Engineering, Civil engineering

## Abstract

For the shallow and circular silo (SCS), when the aspect ratio is between 1.0 and 1.5, the lateral pressure especially dynamic pressure may cause destruction if the size of the silo is large. This paper proposed a modified calculation method of lateral pressure on the silo wall of SCS, considering the elasticities of silo wall and storage materials. The availability of shallow silo and deep silo methods, and the modified method were compared with the experiment and simulation. The results show that the Rankine’s formula is too conservative for the static lateral pressure, and the results of the modified method and Janssen formula are close to that of the experimental and simulation. For the dynamic lateral pressure calculation, Rankine theory is unsafe for the discharging load. The relative error of the dynamic lateral pressure based on Janssen theory is between 20 and 30%, which is too large. The dynamic lateral pressure calculated by the modified method is in good agreement with that of the experimental and simulation, and the relative error is less than 10%. Therefore, the modified method of lateral pressure formula is reasonable, which can provide guidance for the safety design of silo structure.

## Introduction

The characteristics of the silo are large storage, easy mechanization, reasonable mechanics and good anti-seismic performance^1^, which is widely used in grain engineering, chemical engineering, coal engineering, and so on. Especially in China, about one-third of the grain storage structures use silos. Therefore, the safety of the silo is important. It is widely known that concentric discharging at the silo bottom is the most desirable discharging way^2–5^. The idea that the dynamic pressure on the sidewall is greater than the static pressure during concentric discharging has been generally accepted, which may cause the silo’s structural failure and affect the stability of bulk solid handling. Although the silo structure is relatively simple, the storage material is an interactive particle aggregation with complex mechanical properties of solid and liquid. Therefore, the dynamic pressure is the main consideration for the design of the silo. For shallow and circular silo (SCS), when the aspect ratio is between 1.0 and 1.5, the lateral pressure especially the dynamic pressure may destroy the silo if the silo size is large^6,7^. For example, a bolted steel silo that stored 9000 tons of coal ash collapsed after it was first filled due to insufficient consideration of lateral pressure on the silo wall^7^. For the silo with aspect ratio between 1.0 and 1.5, according to silo specification, the lateral pressure on the silo wall should be calculated simultaneously according to the formulas of the shallow silo and the deep silo based on Rankine’s earth pressure theory and Janssen’s theory, respectively, and the maximum value calculated by the above two methods should be adopted^8,9^. However, Rankine’s formula regards the silo wall as smooth, ignoring the friction between bulk material and silo wall, and Janssen’s formula regards the silo as infinite depth and fails to take into account the influence of silo bottom on lateral pressure. Therefore, it is necessary to modify the lateral pressure of SCS whose aspect ratio is between 1.0 and 1.5.

Early silo designers believed that the silo storage pressure was linearly distributed in the direction of height, depending only on the height and density of the silo^10,11^. However, the storage materials are large numbers of particle aggregates, and their mechanical properties are extremely complex, which is difficult to reasonably estimate using the traditional classical mechanics theory and condensed matter physics theory^12^. In recent years, relevant scholars have found that the maximum dynamic pressure on the silo wall is often several times of the static pressure during silo discharging^13–16^. Thus, if the dynamic pressure on silo wall is not considered during the design process, it will lead to the instability of the silo.

Many scholars have paid attentions to the complex dynamic lateral pressure on the silo wall^17–20^. Horabik et al.^17^ studied the mechanical properties of granular materials and their effects on load distribution and discussed the evolution of the constitutive model of granular materials under the mechanical framework. Patel et al.^18^ utilized FEM to analyze the positive pressure, vertical pressure, circumferential stress and longitudinal stress on silo wall under different boundary conditions, considering Drucker-Prager criterion and material properties. Lei et al.^19^ established a new theoretical model of lateral pressure on the silo wall and put forward the empirical expression of stress state transition between quasi-static fluid and quasi-Janssen fluid. An et al.^20^ studied the normal stress characteristics on silo wall by arranging pressure sensors to measure dynamic lateral stress. Unfortunately, the above investigations do not consider the influences of storage materials and silo deformation. Jarrett et al.^21^ considered the effects of interaction between stored material and structure, and studied the stress of material in rectangular flexible silo during filling. Rotter et al.^22^ simulated the flexible wall rectangle silos by finite element method., reached wall elasticity has contribution to the lateral pressure on the silo wall. As a matter of fact, most silos are made of materials with small stiffness, such as metallic plate silos^23,24^. Therefore, when calculating the lateral pressure on the silo wall, it is particularly important to consider the elastic deformations of silo wall and storage material.

In view of this, this paper proposed a modified calculation method of lateral pressure on the silo wall with aspect ratio between 1.0 and 1.5, considering the deformations of silo wall and storage material, which is verified through experimental results and numerical simulation.

## Derivation formula of lateral pressure on the silo wall

### Janssen’s formula and Rankine’s formula

Silos are usually used to store bulk materials such as grains, coals and chemicals. Early silo designers believed that the pressure on the silo wall was linearly distributed in the direction of height, depending only on the height and density of the storage materials in the silo^10,11^. However, as a certain number of particle aggregates, granular materials have complex mechanical properties, which are different from the mechanical characteristics of fluid and solid in the traditional sense. After found this, relevant scholars made appropriate adjustments to the research direction and achieved significant results in granular mechanics. Based on the static equilibrium principle existing in the granular micro-element of bulk storage, Janssen deduced the static lateral pressure on deep silo, which is the reference basis for most national silo design specifications. The pressure on the silo wall calculated by Janssen’s formula is^9^:

1 $$ P_{{\text{h}}} = C_{{\text{h}}} \gamma \rho (1 - e^{{ - \mu {\text{k}}s/\rho }} )/\mu $$

where *μ* is the friction coefficient between the storage materials and silo wall, *ρ* the hydraulic radius of horizontal net section of silo, $s$ the distance from top surface or cone center of gravity to calculated section, *C*_h_ the correction coefficient of horizontal pressure on the silo. When *C*_h_ equals 1.0, Eq. (1) is applicable for the static lateral pressure calculation. While, for the dynamic lateral pressure calculation, *C*_h_ is larger than 1.0.

Rankine’s formula is based on the earth pressure theory to predict the static lateral pressure of shallow silo, which is^9^:

2 $$ P_{{{\text{h1}}}} = {\text{k}}\gamma {\text{s}} $$

where $\gamma$, s and k are the gravity density (kN/m^3^), height (m), and lateral pressure coefficient of storage material, respectively.

The expression of k in Eq. (2) is:

3 $$ {\text{k}} = {\text{tan}}^{2} \left( {\frac{\pi }{4} - \frac{\varphi }{2}} \right) $$

where $\varphi$ is the angle of internal friction of storage materials.

For above two methods, Rankine’s formula ignores the friction between granular material and silo wall, and Janssen’s formula fails to take into account the influence of silo bottom on lateral pressure. At the same time, both the two formulas regard the lateral pressure coefficients as invariant constants. However, in real-life situations, the lateral pressure coefficient varies with the depths of the silo. Therefore, the above two methods for determining the lateral pressure of shallow silo between 1.0 and 1.5 are not reasonable.

### Modification of lateral pressure on the silo wall

**Figure 1:**
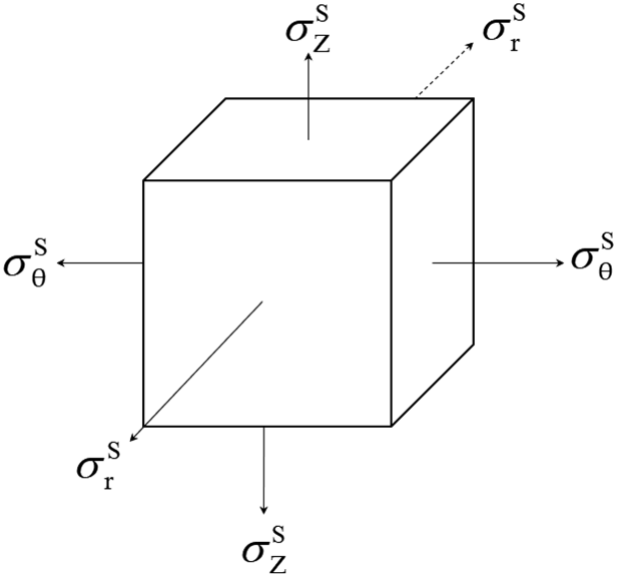
The stress diagram of microelement of storage materials.

According to above analysis, both Rankine’s formula for shallow silo and Janssen’s formula for deep silo do not consider the elastic deformations of the silo and storage materials. According to the theory of plate and shell, the membrane force deforms the silo wall, and the bending moment is almost “zero”. At the bottom of silo, due to the “edge effect”, the bending moment is obvious, when subjected to the action of granular particles^25^. According to the generalized Hooke’s theorem, the stresses in the circumferential, axial and radial directions will affect the circumferential strain. The granular particles near the silo wall are selected as the unit for stress analysis, as shown in Fig. 1.

The superscript (or subscript) “W’’ denotes the silo wall, and the superscript (or subscript) “S” denotes the material stored near the silo wall. The circumferential strain ($\varepsilon_{{\uptheta }}^{{\text{S}}}$) of storage materials is:

4 $$ \varepsilon_{{\uptheta }}^{{\text{S}}} { = }\frac{1}{{E_{{\text{S}}} }}\left[ {\sigma_{{\uptheta }}^{{\text{S}}} - \nu_{{\text{S}}} \left( {\sigma_{{\text{r}}}^{{\text{S}}} + \sigma_{{\text{Z}}}^{{\text{S}}} } \right)} \right] $$

where $\sigma_{{\uptheta }}^{{\text{S}}}$, $\sigma_{{\text{r}}}^{{\text{S}}}$ and $\sigma_{{\text{Z}}}^{{\text{S}}}$ are the circumferential stress, radial stress and axial stress, respectively, $E_{{\text{S}}}$ and $\nu_{{\text{S}}}$ are the Elastic Modulus and Poisson’s ratio of storage materials, respectively.

**Figure 2:**
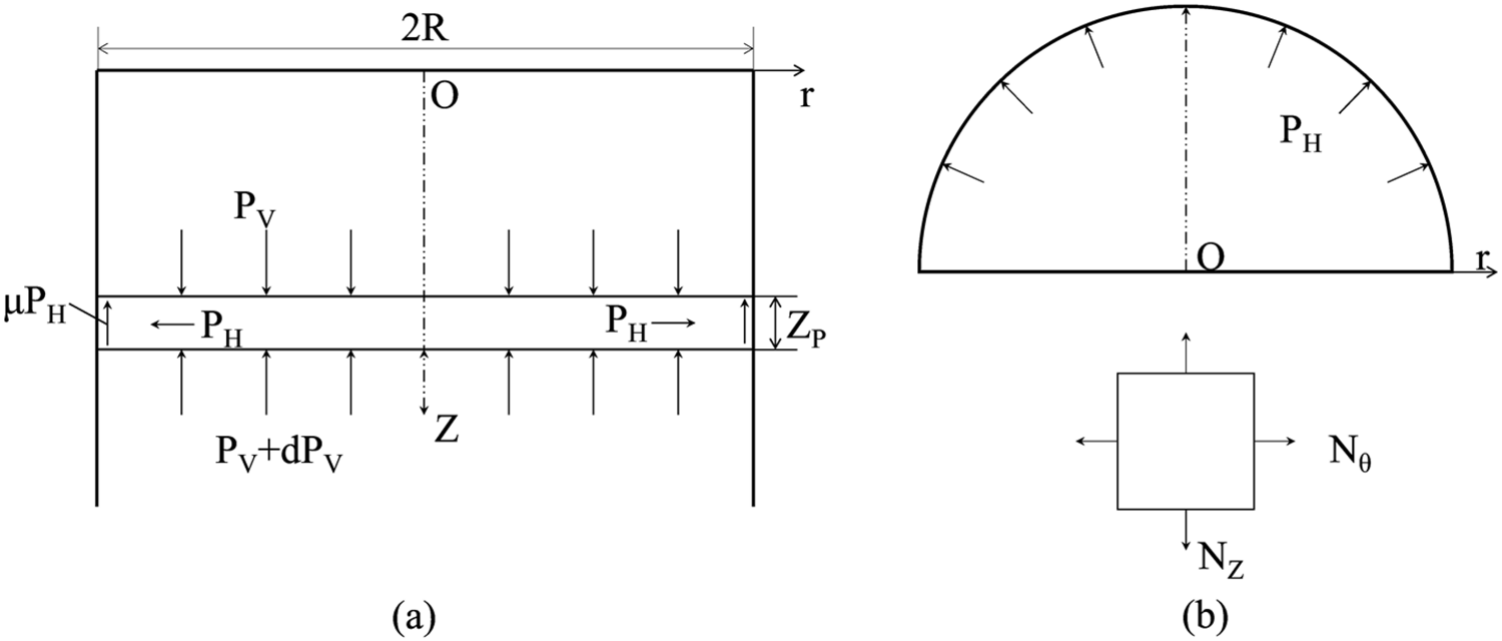
Stress analysis. **(a)** The storage materials granular particles and **(b)** the silo wall.

Since the pressure of storage materials on the silo wall is mainly membrane force, the bending moment is “zero”. According to the elastic mechanics theory, the stress is positive when the storage materials is pulled and is negative when the storage materials is pushed^26^. The axial stress of the element is the negative value of the gravity of storage materials ($P_{{\text{V}}}$).The radial stress ($\sigma_{{\text{r}}}^{{\text{S}}}$) and circumferential stress ($\sigma_{{\uptheta }}^{{\text{S}}}$) are both equal to the negative values of the lateral pressure ($P_{{\text{H}}}$), as shown in Fig. 2.

It is assumed that the pressure and the density of storage materials acting on the cross section of the silo remain unchanged, and the radial deformation of the storage materials near the silo wall is consistent with that of the silo wall^27^. The radial stress and circumferential stress are:

5 $$ \sigma_{{\text{r}}}^{{\text{S}}} { = }\sigma_{{\uptheta }}^{{\text{S}}} { = } - P_{{\text{H}}} $$

6 $$ \sigma_{{\text{Z}}}^{{\text{S}}} { = } - P_{{\text{V}}} $$

Substituting Eqs. (5, 6) into Eq. (4) yields:

7 $$ \varepsilon_{{\uptheta }}^{{\text{S}}} { = }\frac{1}{{E_{{\text{S}}} }}\left[ {\nu_{{\text{S}}} P_{{\text{v}}} + \left( {\nu_{{\text{S}}} - 1} \right)P_{{\text{H}}} } \right] $$

According to the theory of plate and shell, the membrane forces (*N*_Z_ and *N*_θ_) are:

8 $$ N_{{\text{Z}}} { = }\sigma_{{\text{Z}}}^{{\text{W}}} t,\quad N_{{\uptheta }} { = }\sigma_{{\uptheta }}^{{\text{W}}} t $$

where *t* is the thickness of silo wall.

Taking the semi-circular ring of silo wall as the research object, the equilibrium equations in vertical direction and horizontal direction are:

9 $$ N_{{\uptheta }} { = }P_{{\text{H}}} R $$

10 $$ N_{{\text{Z}}} { = } - \int_{0}^{{\text{Z}}} {\mu P_{{\text{H}}} } dZ $$

In addition, according to the theory of plate and shell, the circumferential strain is:

11 $$ \varepsilon_{{\uptheta }}^{{\text{W}}} { = }\frac{1}{{E_{{\text{W}}} }}\left[ {\sigma_{{\uptheta }}^{{\text{W}}} - \nu_{{\text{W}}} \sigma_{{\text{Z}}}^{{\text{W}}} } \right]{ = }\frac{1}{{E_{{\text{W}}} t}}\left[ {N_{{\uptheta }} - \nu_{{\text{W}}} N_{{\text{Z}}} } \right] $$

Since the circular silo is an axisymmetric structure, circumferential strain can be calculated as:

12 $$ \varepsilon_{{\uptheta }} { = }{{u_{{\text{r}}} } \mathord{\left/ {\vphantom {{u_{{\text{r}}} } r}} \right. \kern-\nulldelimiterspace} r} $$

where $u_{{\text{r}}}$ is radial displacement.

Because the radial deformation of the silo wall is the same as that of the granular particles near silo wall, the strains are the same for the silo wall and the granular particles, which yields

13 $$ u_{{\text{r}}}^{{\text{W}}} { = }u_{{\text{r}}}^{{\text{S}}} ,\varepsilon_{{\uptheta }}^{{\text{W}}} { = }\varepsilon_{{\uptheta }}^{{\text{S}}} $$

Based on Eqs. (7) and (11), the following equals is satisfied:

14 $$ \frac{1}{{E_{{\text{S}}} }}\left[ {\nu_{{\text{S}}} {\text{P}}_{{\text{V}}} + \left( {\nu_{{\text{S}}} - 1} \right)P_{{\text{H}}} } \right]{ = }\frac{1}{{E_{{\text{W}}} t}}\left[ {N_{{\uptheta }} - \nu_{{\text{W}}} N_{{\text{Z}}} } \right] $$

Substituting Eqs.  (9) and (10) into Eq. (14), one can obtain that:

15 $$ \nu_{{\text{S}}} P_{{\text{V}}} { = }\left( {1 - \nu_{{\text{S}}} { + }\frac{{E_{{\text{S}}} R}}{{E_{{\text{W}}} t}}} \right)P_{{\text{H}}} { + }\frac{{E_{{\text{S}}} R}}{{E_{{\text{W}}} t}} \times \frac{{\mu v_{{\text{W}}} }}{R}\int_{0}^{{\text{Z}}} {P_{{\text{H}}} dZ} $$

where $\eta { = }{{E_{{\text{S}}} R} \mathord{\left/ {\vphantom {{E_{{\text{S}}} R} {E_{{\text{W}}} }}} \right. \kern-\nulldelimiterspace} {E_{{\text{W}}} }}t$ is the stiffness ratio, and $v_{{\text{W}}}$ is a constant less than 1.0. For general metallic silos, the range of stiffness ratio lies between 0.01 and 0.2.

Dividing both sides of Eq. (15) by *P*_H_, simultaneously, the second term on the right side of Eq. (15) is approximately zero, and Eq.  (14) can be rewritten as:

16 $$ {\text{k}}_{1} { = }\frac{{{\text{P}}_{{\text{H}}} }}{{{\text{P}}_{{\text{v}}} }}{ = }\frac{{\nu_{{\text{S}}} }}{{1 - \nu_{{\text{S}}} + \eta }} $$

Equation (16) is the correction coefficient of lateral pressure considering the elasticity of silo wall.

For the concrete silo with rigid wall, assuming that the granular particles in the silo are elastic and axisymmetric, the stress–strain relationship of the silo is:

17 $$ \left[ \begin{gathered} \sigma_{{\text{r}}}^{{\text{S}}} { = }\Delta \left( {\varepsilon_{{\text{r}}}^{{\text{S}}} + \frac{\nu }{{1 - \nu_{{\text{S}}} }}\varepsilon_{{\uptheta }}^{{\text{S}}} { + }\frac{\nu }{{1 - \nu_{{\text{S}}} }}\varepsilon_{{\text{Z}}}^{{\text{S}}} } \right) \hfill \\ \sigma_{{\uptheta }}^{{\text{S}}} { = }\Delta \left( {\frac{\nu }{{1 - \nu_{{\text{S}}} }}\varepsilon_{{\text{r}}}^{{\text{S}}} + \varepsilon_{{\uptheta }}^{{\text{S}}} { + }\frac{{\nu_{{\text{S}}} }}{{1 - \nu_{{\text{S}}} }}\varepsilon_{{\text{Z}}}^{{\text{S}}} } \right) \hfill \\ \sigma_{{\text{Z}}}^{{\text{S}}} { = }\Delta \left( {\frac{\nu }{{1 - \nu_{{\text{S}}} }}\varepsilon_{{\text{r}}}^{{\text{S}}} + \frac{\nu }{{1 - \nu_{{\text{S}}} }}\varepsilon_{{\uptheta }}^{{\text{S}}} { + }\varepsilon_{{\text{Z}}}^{{\text{S}}} } \right) \hfill \\ \sigma_{{{\text{rZ}}}}^{{\text{S}}} { = }\Delta \left( {\frac{{1 - 2\nu_{{\text{S}}} }}{{2\left( {1 - \nu_{{\text{S}}} } \right)}}\varepsilon_{{{\text{rZ}}}}^{{\text{S}}} } \right) \hfill \\ \end{gathered} \right. $$

where $$\Delta { = }\frac{{E_{{\text{S}}} \left( {1 - \nu_{{\text{S}}} } \right)}}{{\left( {1{ + }\nu_{{\text{S}}} } \right)\left( {1 - 2\nu_{{\text{S}}} } \right)}}$$.

Because of the self-weight of granular particles, $\varepsilon_{{\uptheta }}^{{\text{S}}}$ and $\varepsilon_{{\text{r}}}^{{\text{S}}}$ are much larger than $\varepsilon_{{\text{Z}}}^{{\text{S}}}$, the following equation can be obtained based on Eq. (17).

18 $$ \left\{ \begin{gathered} \sigma_{{\text{r}}}^{{\text{S}}} \approx \frac{{E_{{\text{S}}} \nu_{{\text{S}}} }}{{\left( {1 + \nu_{{\text{S}}} } \right)\left( {1 - 2\nu_{{\text{S}}} } \right)}}\varepsilon_{{\text{r}}}^{{\text{S}}} \hfill \\ \sigma_{{\text{Z}}}^{{\text{S}}} \approx \frac{{E_{{\text{S}}} \left( {1 - \nu_{{\text{S}}} } \right)}}{{\left( {1 + \nu_{{\text{S}}} } \right)\left( {1 - 2\nu_{{\text{S}}} } \right)}}\varepsilon_{{\text{Z}}}^{{\text{S}}} \hfill \\ \end{gathered} \right. $$

Considering the elastic state of the storage material in the silo, the lateral pressure coefficient ${\text{k}}_{2}$ is approximately:

19 $$ {\text{k}}_{2} { = }\frac{{\sigma_{{\text{r}}}^{{\text{S}}} }}{{\sigma_{{\text{Z}}}^{{\text{S}}} }} \approx \frac{{\nu_{{\text{S}}} }}{{1 - \nu_{{\text{S}}} }} $$

For the concrete silo with rigid wall, $\eta \approx 0$ because of $E_{{\text{W}}} t \gg E_{{\text{S}}} R$. Therefore, when $\eta = 0$, Eq. (19) is actually the same as Eq. (16).

Taking Eq. (16) into Eq. (3), the formula for calculating the lateral pressure considering the elasticities of silo wall and bulk particles can be written as

20 $$ P_{{\text{h}}} = C_{{\text{h}}} \gamma \rho (1 - e^{{ - \mu {\text{k}}_{1} s/\rho }} )/\mu $$

For shallow silos with aspect ratio between 1.0 and 1.5, Eq. (20) is supposed to be applicable to calculate the lateral pressure. In the following, this paper will utilize the experimental and numerical simulation to compare the above modified lateral pressure calculation by Eq. (20) with the results by Rankine’s and Janssen’s formulas. Based on silo specification^9^, the parameters in the above equations are taken as: *γ* = 8 kN/m^3^, *ρ* = 0.25, *μ* = 0.4, *φ* = 25°, k = 0.4058, k_1_ = 0.333, *C*_h0_ = 1.8, $E_{{\text{W}}} { = }3 \times 10^{6}$ and $\nu_{{\text{S}}} { = }0.3$, during the theoretical calculation.

## Results and discussions

### Experiment verification

Based on the National Grain Storge in the eastern part of Zhengzhou City, Henan Province, China, the newly-built silos are reinforced concrete shallow circular silos. The diameter(D) of each silo is 28 m, and the height is 36.4 m. The circular shallow silo is well representative. According to the similarity theory^28^, the model silo is made of 1:28 ratio reduction of the real-life silo, resulting the diameter and height are 1.0 m and 1.3 m, and the aspect ratio is 1.3. The funnel is made of steel, with a diameter of 0.1 m and an inclination angle of 45^°^.

**Figure 3:**
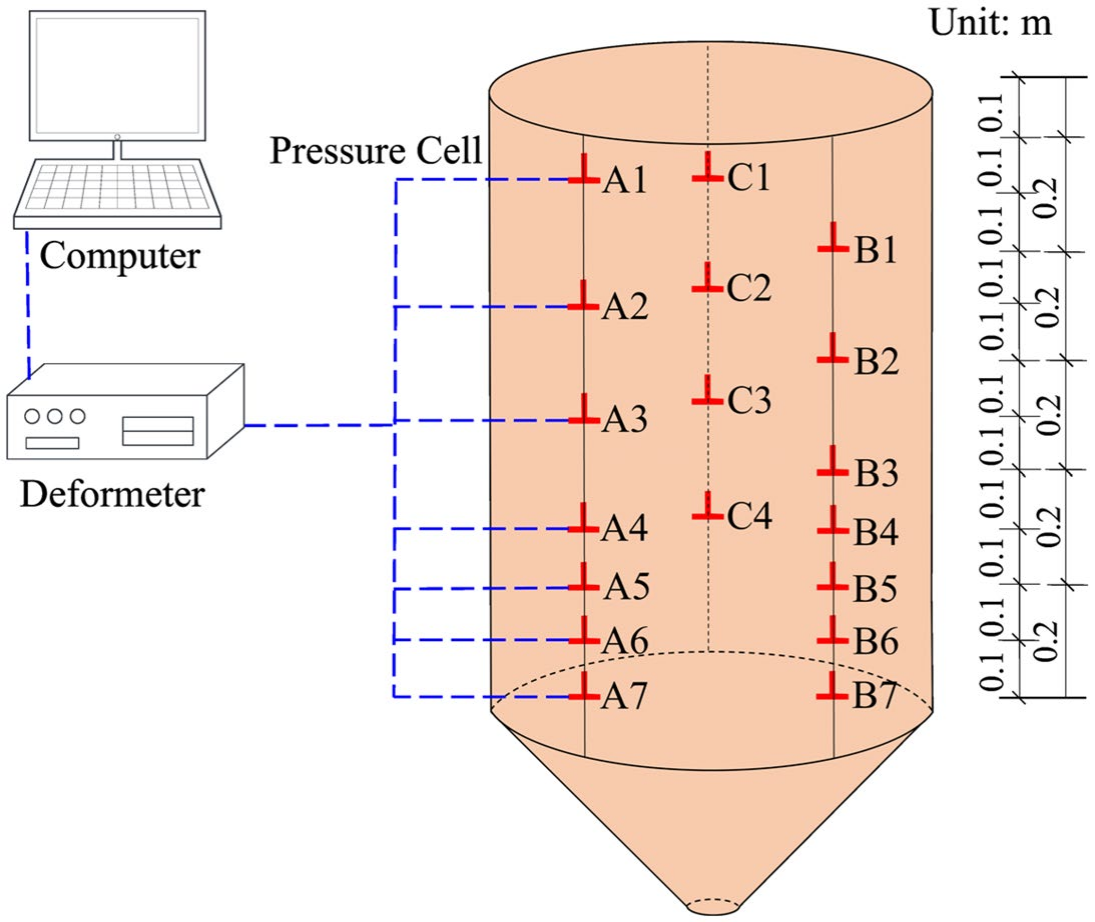
Schematic diagram of experiment system.

Since the organic glass silos are convenient to observe the flow patterns of storage materials and to study the wall normal stresses during silo discharging^14,29–33^, the organic glass, whose elastic modulus(*E*) and Poisson’s ratio(*v*) are 2.758 × 10^3^ (MPa) and 0.29, respectively^29^, is selected to make the model silo. Three columns of sensors A, B and C are arranged along the height direction of the silo wall. The experimental system is shown in Fig. 3.

The storage materials are wheat, whose physical parameters are given in Table 1^9^. Under the same experiment conditions, silo filling and silo discharging are both conducted three times, and the average value of experimental results is adopted.

**Table 1:** Physical parameters of wheat.

Storage materials	Modulus of elasticity (GPa)	Poisson’s ratio	Density (kg/m^3^)	Angle of repose (^o^)	Cohesion (kPa)	Internal friction angle (^o^)	Angle of dilatancy (^o^)	Coefficient of friction
Wheat	3.0	0.3	816	25	300	25	17.6	0.4

**Figure 4:**
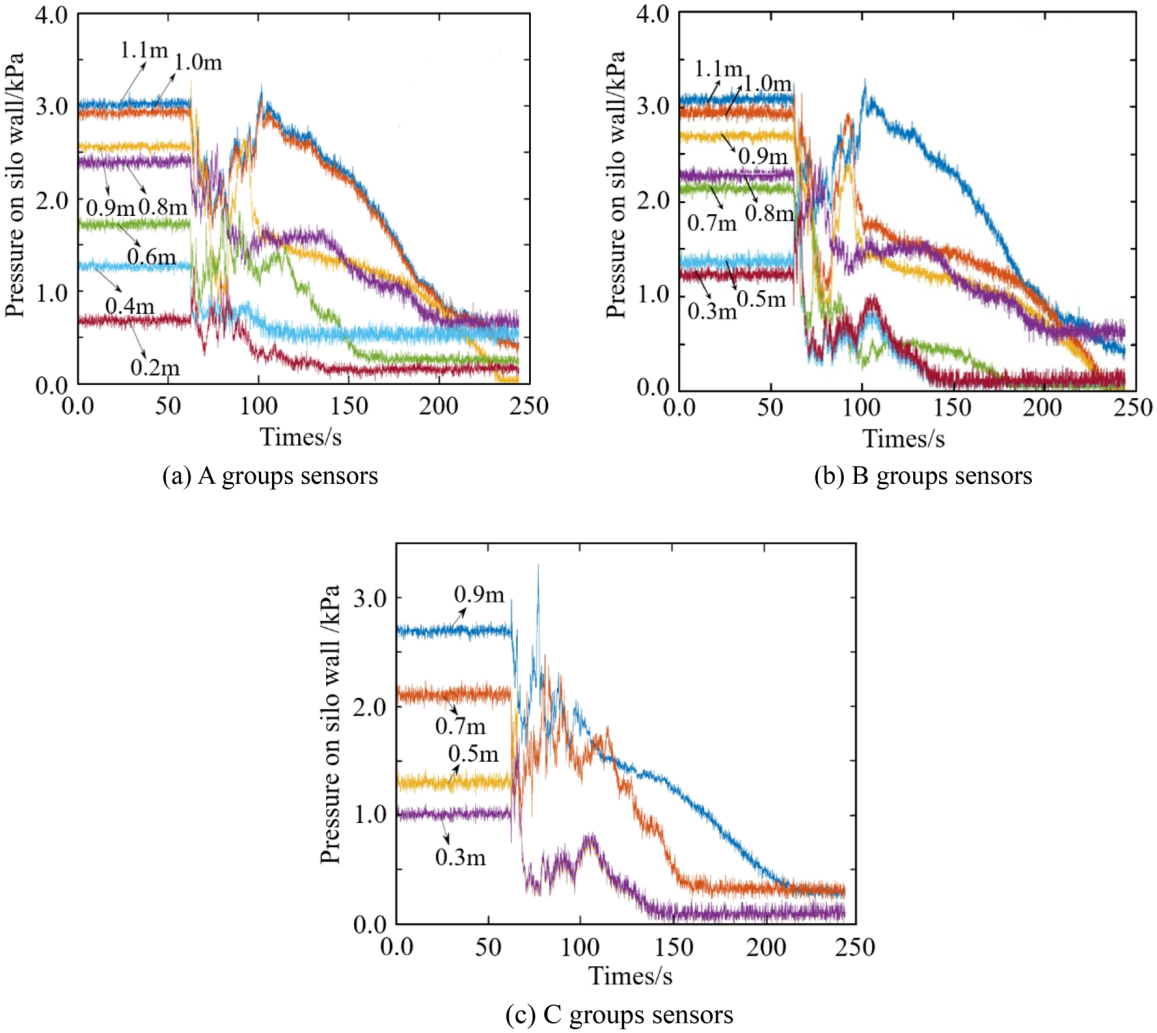
Experiment results.

The results from three groups of soil pressure sensors are shown in Fig. 4. From 0 to 62 s, the storage materials in the silo are static, and the pressure corresponding to the horizontal curves with weak oscillation is the static lateral pressure. After 62 s, the silo discharging begins. Although the stacking height of the storage materials is decreasing, the pressure on the silo wall suddenly increases. When the silo discharging is completed, the curves tend to be stable. The maximum pressure occurs at the initial stage of silo discharging, while the dynamic lateral pressure at the end of the discharging is relatively small. The above results are in accord with the references^34–39^, which shows that the designed experiment is reliable.

**Figure 5:**
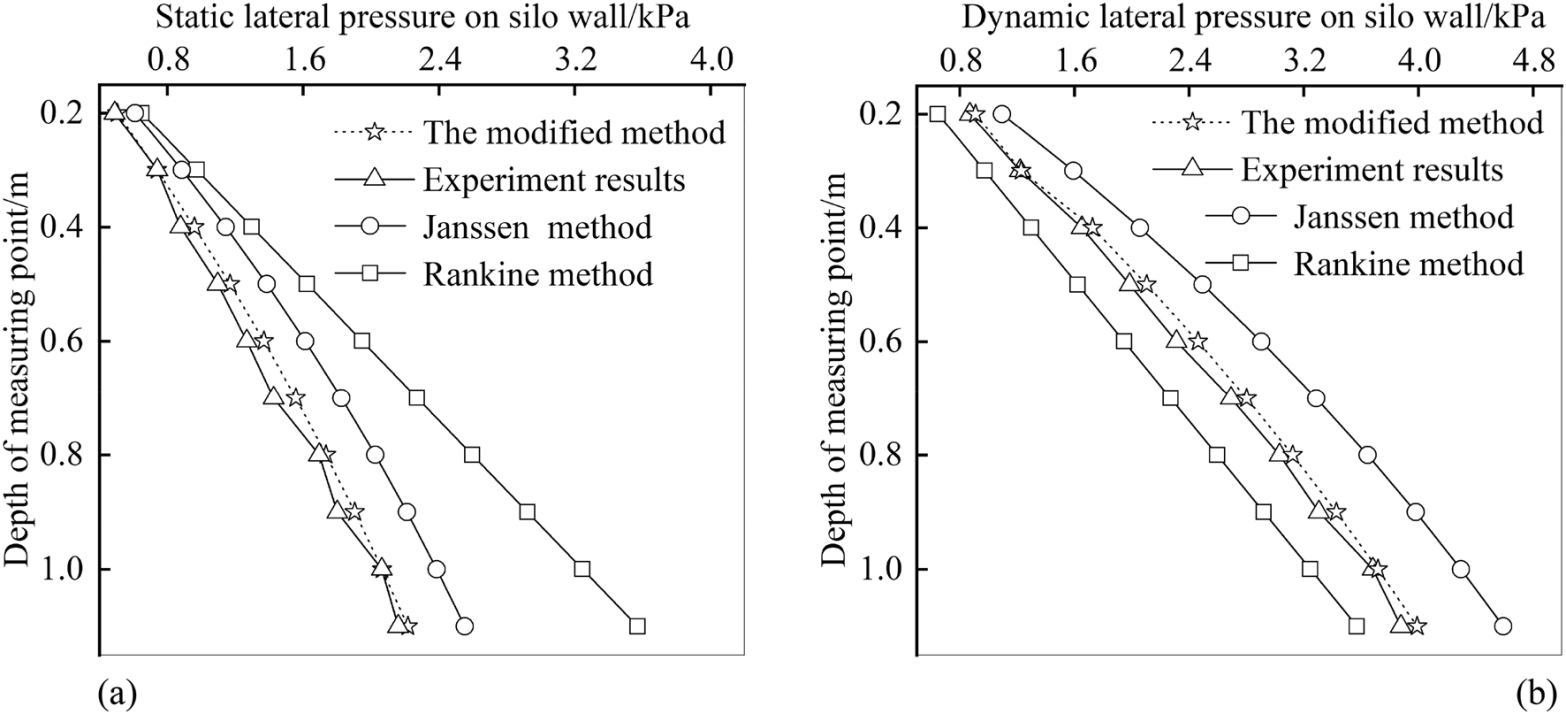
Comparisons of the theoretical and the experimental results: **(a)** the static and **(b)** the dynamic pressure on the silo wall.

Accordingly, the comparisons of theoretical and the experimental results are shown in Fig. 5. It can be seen from Fig. 5a that for the static lateral pressure, the theoretical calculation values at different depths of measuring points are all greater than the experimental values. Therefore, the three theoretical calculation methods all satisfy the safety requirements. However, the maximum value calculated by Rankine’s formula is 1.6 times of the experiment and 1.4 times of Janssen’s formula. It is too conservative to determine the static lateral pressure according to the specification. The values calculated by the modified method are close to that of Janssen’s formula at different depths of measuring points. Therefore, the modified method and Janssen’s formula is more suitable for static lateral pressure calculation. It can be seen from Fig. 5b that for the dynamic lateral pressure, the values calculated by Janssen’s formula are much larger than the experimental value, and the experimental values at all measuring points are greater than the values calculated by Rankine’s formula. Therefore, for the dynamic lateral pressure, it is unsafe to calculate the pressure according to the Rankine’s formula of shallow circular silo, and it is too conservative to calculate the pressure according to the Janssen’s formula of deep silo. By contrast, the results of the modified method are close to and larger than that of the experiments, which is accurate and guarantees the design security at the same time. In addition, for Janssen’s formula, the relative error of each measuring point is between 20 and 30%, which is too large. According to the modified method, the relative error of each measuring point is less than 10%. Therefore, the modified method is the most reliable.

### Numerical simulation verification

The investigations about shallow silos with aspect ratios between 1.0 and 1.5 are rarely reported, especially in the experiments. In order to better verify the applicability of the proposed method, numerical simulation was used to analyze the simulated silo with aspect ratios of 1.1, 1.3 and 1.42. The simulation results with an aspect ratio of 1.3 were compared with the above experimental results to verify the accuracy of the numerical model. silo models were established using the particle flow code 2D (PFC^2D^). In PFC^2D^, The discrete element model established in this paper adopts the linear contact model, Using fish language for command operations. Wall command is mainly used to build silo wall, and ball command is used to generate particles. For PFC^2D^ simulation, it is important to determine the contact stiffness of wall and particle. To obtain the reasonable contact stiffness, a reference value of the contact stiffness of the wall and particles is obtained by using the calculation formula of the contact stiffness, and then the model is repeatedly calibrated through the test results to determine the final value^39^. The detailed calculation steps are as follows: Firstly, the reference values of normal and tangential stiffness of particles are obtained by theoretical calculation. Then, the control variable is used to adjust the reference value continuously, so that the simulated lateral pressure, experimental results and specification values are consistent. Finally, the accurate values of normal and tangential stiffness of particles are obtained. The simulation parameters are shown in Table 2. The particle diameter of wheat in the test is about 0.003–0.005 m, so in this simulation, the particle radius is 0.002 m, and all particle radiuses remain the same. In addition, the particle density was 816 kg/m^3^, the same as wheat.

**Table 2:** Parameters of numerical simulation of silo.

Normal stiffness of silo wall (N/m)	Tangential stiffness of silo wall (N/m)	Normal stiffness of storage materials (N/m)	Tangential stiffness of storage materials (N/m)	Coefficient of internal friction	Coefficient of external friction
4 × 10^6^	2 × 10^6^	1.3 × 10^5^	1.0 × 10^5^	0.49	0.3

**Figure 6:**
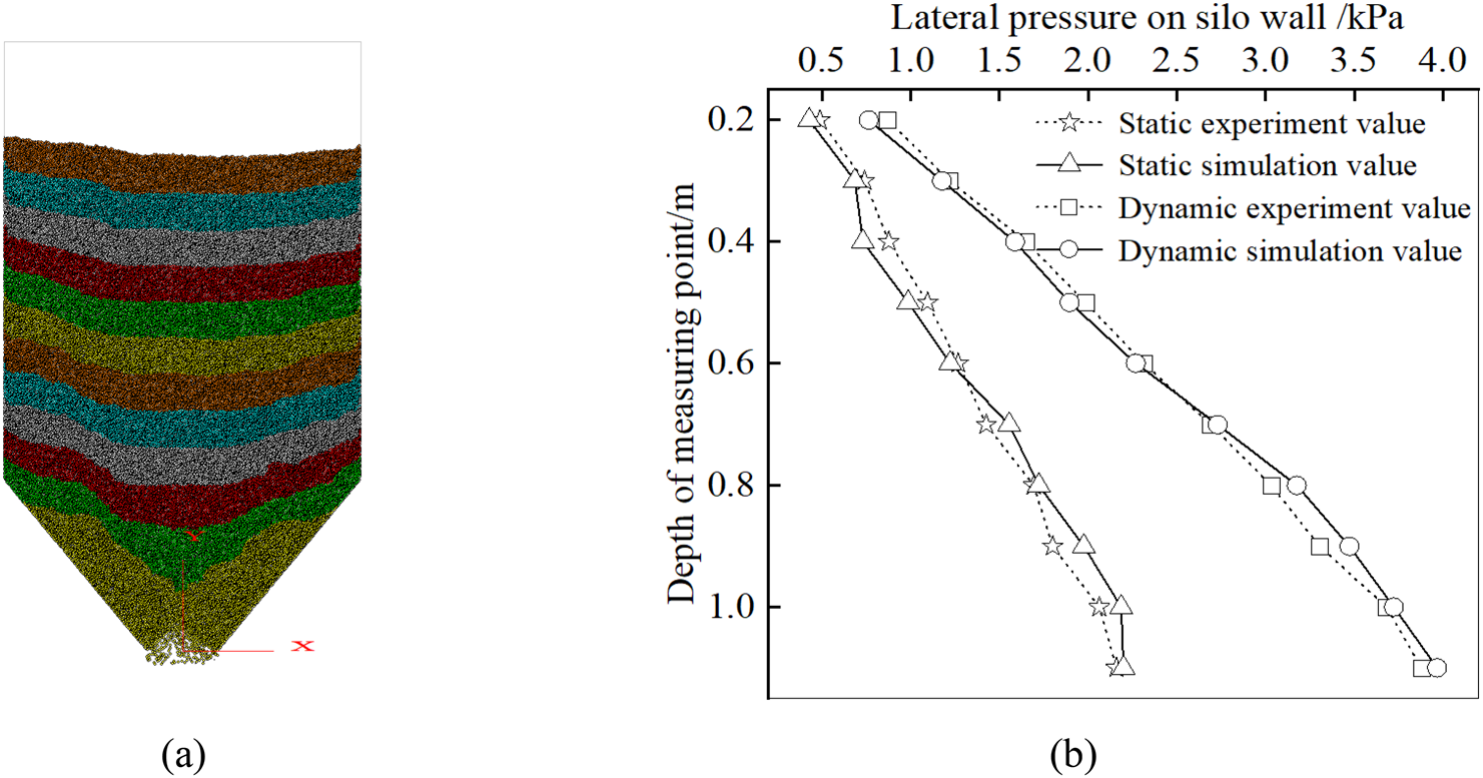
**(a)** Simulation model and **(b)** result analysis.

The storage particles are generated in layers in the silo using the create ball function, and the unbalanced force is eliminated using the quit and solve functions, to make the storage particles in the silo reach static equilibrium. Then, the results from the silo with aspect ratio of 1.3 are compared with the above experimental results to verify the accuracy of the numerical simulation. The simulation model of silo with an aspect ratio of 1.3 are shown in Fig. 6a. The comparisons of the experiment and simulation are presented in Fig. 6b. From Fig. 6b, it is seen both the static pressure and the dynamic pressure values in simulation match perfectly with the experiment results, which verifies the reliability of the numerical simulation.

**Figure 7:**
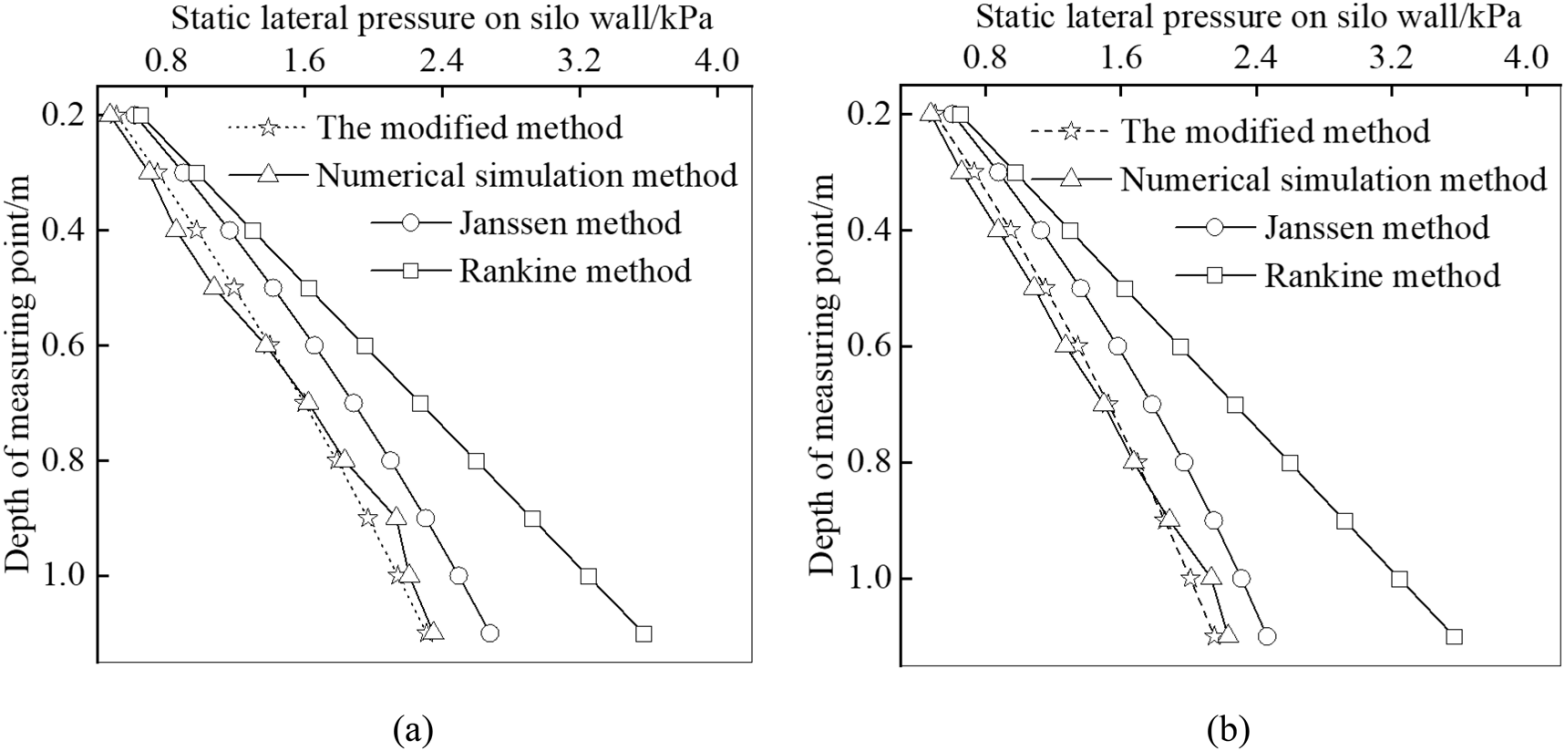
Comparison of theoretical values and numerical simulation values of static lateral pressure on soil with aspect ratio of **(a)** 1.1 and **(b)** 1.42.

For the static lateral pressure on the silos with different aspect ratios, the results of the modified method, Janssen’s formula, Rankine’s formula and the simulation are compared, as shown in Fig. 7. It can be found that the simulation values are in good agreement with that calculated by the modified method for soil with aspect ratios of 1.1 and 1.42, and the fluctuation is very small.

**Figure 8:**
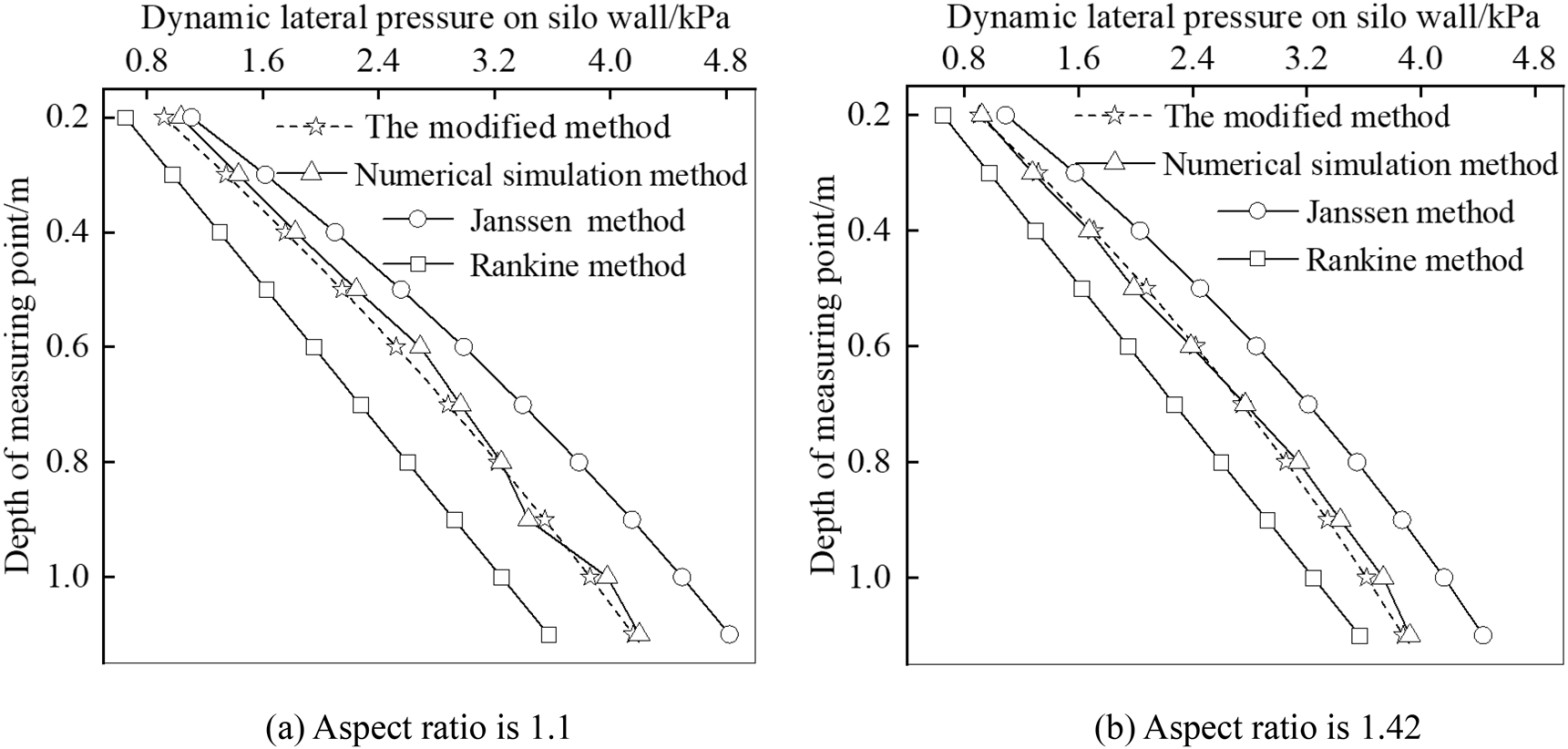
Comparisons of theoretical values and numerical simulation values of dynamic lateral pressure.

For the dynamic lateral pressure on the silos with different aspect ratios, calculations by the above theory and the simulation are compared in Fig. 8. It can be seen that the values calculated by Rankine do not meet the design requirements, no matter the aspect ratio is 1.1 or 1.42. The values calculated by the modified method are in good agreement with that by simulation for soil with aspect ratios of 1.1 and 1.42.

In summary, the modified method, which considering the elastic deformations of silo wall and storage materials, is valid for predicting the lateral pressure on the silos with aspect ratio between 1.0 and 1.5, and thus is recommended for the design of the shallow silos with aspect ratio between 1.0 and 1.5.

However, the experimental data obtained in this paper are still too small to get statistical conclusions, and more silo tests are needed. It is necessary to consider the influences of storage materials and silo wall materials on the lateral pressure, especially the change caused by the increase of diameter and depth. It is undeniable that further experimental data are needed to validate the proposed model. Because in such a complex system, real test data is very critical to verify the applicability of the proposed model, which is also an indispensable step. Statistical conclusions are not possible until more experimental data are available.

In addition, the calculation method of this paper is a preliminary exploration, although under limited conditions, the calculated results are in good agreement with the test results and simulation results, it cannot represent in actual engineering. The proposed formula is only a preliminary work and has certain limitations. It is hoped that more scholars will conduct more further research on this calculation method in the future.

## Conclusions

This paper analyzed the shortcomings of the lateral pressure calculation of shallow circular silo with aspect ratio between 1.0 and 1.5 in the specification based on Rankine and Janssen theories. Considering the influence of elastic deformations of silo wall and storage materials, a modified method for calculating lateral pressure was proposed for the design of silos with aspect ratio between 1.0 and 1.5. The effectiveness of this method is verified by experiments and numerical simulations. The main conclusions are as follow:

(1) When checking the load under silo discharging, the dynamic pressure calculation of shallow circular silo based on Rankine theory is not safe. While the calculation of deep silo based on Janssen theory is conservative, and the relative error of each measuring point is between 20 and 30%, which is too large.

(2) The static and dynamic results based on the proposed modified method are both in good agreement with the experimental and numerical simulations. It is proved that the modified lateral pressure calculation method is a good choice for shallow silos with aspect ratio between 1.0 and 1.5, which can provide a reliable theoretical basis for further improving the design of silo.
